# A novel model fitted to multiple life stages of malaria for assessing efficacy of transmission-blocking interventions

**DOI:** 10.1186/s12936-017-1782-3

**Published:** 2017-04-04

**Authors:** Ellie Sherrard-Smith, Thomas S. Churcher, Leanna M. Upton, Katarzyna A. Sala, Sara E. Zakutansky, Hannah C. Slater, Andrew M. Blagborough, Michael Betancourt

**Affiliations:** 1 MRC Centre for Outbreak Analysis and Modelling, Department of Infectious Disease Epidemiology, Imperial College London, Norfolk Place, London, W2 1PG UK; 2 Department of Life Sciences, Imperial College London, South Kensington, London, SW7 2AZ UK; 3 Department of Statistics, University of Warwick, Warwick, UK

**Keywords:** Atovaquone, *Plasmodium berghei*, Transmission-blocking drugs, Transmission-blocking vaccines

## Abstract

**Background:**

Transmission-blocking interventions (TBIs) aim to eliminate malaria by reducing transmission of the parasite between the host and the invertebrate vector. TBIs include transmission-blocking drugs and vaccines that, when given to humans, are taken up by mosquitoes and inhibit parasitic development within the vector. Accurate methodologies are key to assess TBI efficacy to ensure that only the most potent candidates progress to expensive and time-consuming clinical trials. Measuring intervention efficacy can be problematic because there is substantial variation in the number of parasites in both the host and vector populations, which can impact transmission even in laboratory settings.

**Methods:**

A statistically robust empirical method is introduced for estimating intervention efficacy from standardised population assay experiments. This method will be more reliable than simple summary statistics as it captures changes in parasite density in different life-stages. It also allows efficacy estimates at a finer resolution than previous methods enabling the impact of the intervention over successive generations to be tracked. A major advantage of the new methodology is that it makes no assumptions on the population dynamics of infection. This enables both host-to-vector and vector-to-host transmission to be density-dependent (or other) processes and generates easy-to-understand estimates of intervention efficacy.

**Results:**

This method increases the precision of intervention efficacy estimates and demonstrates that relying on changes in infection prevalence (the proportion of infected hosts) alone may be insufficient to capture the impact of TBIs, which also suppress parasite density in secondarily infected hosts.

**Conclusions:**

The method indicates that potentially useful, partially effective TBIs may require multiple infection cycles before substantial reductions in prevalence are observed, despite more rapidly suppressing parasite density. Accurate models to quantify efficacy will have important implications for understanding how TBI candidates might perform in field situations and how they should be evaluated in clinical trials.

**Electronic supplementary material:**

The online version of this article (doi:10.1186/s12936-017-1782-3) contains supplementary material, which is available to authorized users.

## Background

Malaria remains a serious public health concern; in 2015, an estimated 214 million (range 149–303 million) new cases and 438,000 (236,000–635,000) deaths resulted from the infection [1]. Malaria is transmitted between vertebrate hosts by anopheline mosquitoes. Circulating male and female gametocytes are ingested by mosquitoes during blood feeding where they undergo sexual reproduction before developing into oocysts on the wall of the midgut. On rupturing, an average 1250 (interquartile range 313–2400) salivary gland sporozoites are released from a single *Plasmodium falciparum* oocyst [2]. Sporozoites are then injected onward to the vertebrate host during a subsequent feed. Within the host, sporozoites travel to the liver and invade cells, divide and proliferate to produce tens of thousands of haploid forms, merozoites, per liver cell. Merozoites go on to reproduce asexually, resulting in blood-stage parasites. These can differentiate into gametocytes that complete the life cycle when transmitted back to the insect vector.

There are multiple transmission-blocking interventions (TBIs) currently under development that aim to reduce transmission by inhibiting the development of oocysts in the mosquito midgut or by targeting sexual, sporogonic and/or mosquito antigens (SSM-VIMT) [3]. Different assays have been developed to capture the data required to evaluate the efficacy of different TBIs and triage the most promising candidates [4]. The current gold standard is the standard membrane feeding assay (SMFA) [5, 6]. Here, mosquitoes are fed on cultured, infected blood through a membrane and oocysts can develop. Additionally, multiple derivative assays such as the direct membrane feeding assay (DMFA) and the direct (skin) feeding assay (DFA) are used [5]. The efficacy of drugs and vaccines against malaria have been assessed using data from these assays by measuring the relative reduction in the mean number or prevalence of oocysts in the vector, or prevalence for treated host populations in comparison to control groups [7, 8]. The highly aggregated (overdispersed) distribution of oocysts means that analysis of the reduction in the mean count of oocysts is inherently uncertain [9] and large sample sizes are needed to accurately capture infections [5]. One approach to overcome this is the use of the zero-inflated negative binomial distribution [10] of parasitic life stages that has been used to assess vaccines [11], and suggested as an improvement to capture the influence of aggregated infections on efficacy estimates [9].

A second concern for TBI assessment has been that although SMFA (or its derivatives) capture reductions in infections to mosquitoes, they fail to capture the subsequent onward infectivity to the human host or the onward transmission over multiple cycles. To do this in laboratory studies, multi-generational experiments have been developed that simulate the passage of *Plasmodium berghei* between populations of mice and *Anopheles stephensi* mosquitoes [12, 13]. Using this approach, the transmission-blocking drug atovaquone (ATV), titrated to give a 32% reduction in oocyst intensity (ATV-32%) in a DFA, was found to have an estimated effect size of 20.4% [12]. The effect size measures the ability of a treatment to reduce the basic reproduction number of the parasite (the average number of secondary infections caused by an infected host). These estimates were made using a chain-binomial model [14, 15], but the assumptions inherent in the model affect estimation and need further validation. Firstly, it assumes that all infectious mosquitoes and mice are equally infectious [15]. (In fact, the probability of infection from vertebrates-to-vectors or vice versa increases when hosts have higher infection intensities [16].) Secondly, the number of parasites in successive life stages is highly variable; for example, mosquitoes biting on the same blood source are likely to have widely differing oocyst or sporozoite counts [17]. The importance of parasite intensity on transmission means that it is necessary to account for stochastic variability. Thirdly, the chain binomial model assumes that the probabilities of infection from multiple infectious bites are independent. Finally, although the reproduction number and effect size calculations are widely understood by mathematical modellers, these terms are not generally used by those working in TBI development who tend to measure efficacy as either the transmission-blocking activity ((TBA), the reduction in the number of infected hosts or vectors) or transmission-reduction activity [(TRA), the reduction in mean parasite intensity of the host or vector population]. To overcome these issues, a probabilistic Bayesian model is developed that captures the generative structure of the complex data. This makes it possible to capture the relationship between successive stages of malaria across multiple vertebrate-to-vertebrate transmission cycles and generate TBA and TRA estimates. The zero-inflated negatively binomially distributed data for each parasitic life stage is used to inform the transmission probabilities between vector and host or host and vector. In this way, the transmission probability of a more heavily infected vector or host population (where each individual, on average, harbours a relatively high number of parasitic stages) could be higher than one with lower density infections even where prevalence (the proportion of individuals in the population with any infection) is matched. The Bayesian approach incorporates the variability between transmission events, which is much harder to incorporate into frequentist approaches without relying on approximations. This allows the uncertainty in the system to be captured in the model and enables relatively precise estimates of efficacy (the percentage reduction in parasite prevalence or infection intensity achieved by the TBI) to be broken down by transmission cycle and biting group (the number of potentially infectious mosquitoes biting each mouse during a transmission cycle) to provide a more detailed analysis of data.

## Methods

### Experimental design

**Fig. 1:**
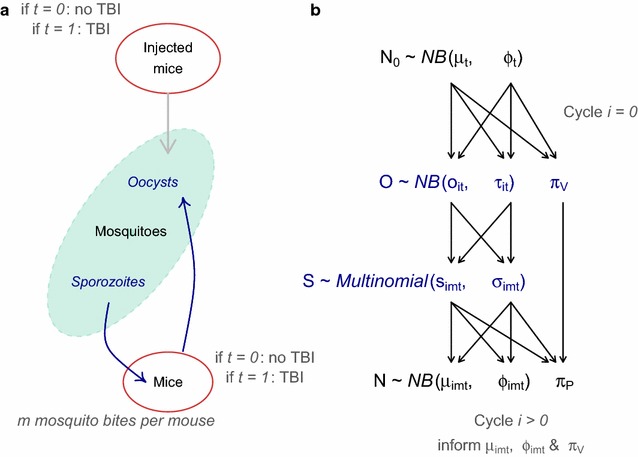
A graphical outline of the multi-generational transmission experiment (**a**) and its mathematical representation (**b**). Ten days prior to treatment, 5 female TO mice (6–8 weeks old) were injected with 10^7^–10^8^ *Plasmodium berghei* clone ANKA 2.34. One day prior to the trial, mosquitoes were starved. Infected mice were either treated with the transmission-blocking intervention, atovaquone ATV (0.5 µg kg^−1^ in 100 µl dimethyl sulfoxide DMSO), or given a negative control (DSMO alone). After 2 h, mice were anaesthetized and 500 naïve *An. stephensi* (line *sd* 500) mosquitoes were allowed to feed on all 5 mice simultaneously and at random so any mosquito could feed on any mouse. This cohort of 500 mosquitoes was sub-sampled (*n* = up to 50 mosquitoes) and dissected 10 days after feeding to measure the prevalence and intensity of oocysts (*O’*). The mosquitoes were at their most infectious 21 days after feeding on the infected mice [8]. At this point, a group of 5 naïve mice (*N*) were anaesthetized and fed to a specified number of mosquitoes (*m* = 1–5 mosquito bites per mouse). Immediately after successful feeding (determined by an engorged abdomen), the sporozoites (*S*) remaining within the mosquito post-feeding were then scored on a binned scale (representing either 0, 1–10,11–100, 101–1000, or 1000 + sporozoites per mosquito). Over 10 days, the bite-exposed mice were sampled for parasitaemia and gametocytaemia. These mice were then treated with the control or ATV and a new cohort of 500 naïve mosquitoes were allowed to feed on any mouse simultaneously at the start of the second transmission cycle (*i* = 2 cycles). The transmission cycles were repeated four times. This experiment was reported previously in [12]. **b** The probabilistic Bayesian model mirrored the experimental set up. The initial parasite density generated by injecting mice *N*_0_, the oocyst intensity *O’* and the parasite density in mice transmitted by mosquito bites *N*, are modelled assuming zero-inflated negative binomial distributions. The sporozoite count *S* data for the biting mosquitoes were censored which means that the data are modelled as a multinomial distribution. These distributions are defined by the mean (*µ*, *o*, *s* for parasite density in mice, oocyst counts and sporozoite counts in mosquitoes) and dispersion (*φ, τ, σ* for parasite density in mice, oocyst counts and sporozoite counts in mosquitoes) and zero-inflation (*π*_*P*_ *, π*_*V*_) parameters. Parameters from the previous life stage are used to inform the next (the respective parameter informing the subsequent life-stage is indicated by the* arrows*). The biting effect *m* is modelled when sporozoites in mosquitoes propagate parasite infections in mice for each transmission cycle *i* and treatment arm *t*. All care and handling of animals strictly followed the Guidelines for Animal Care and Use prepared by Imperial College London, and was performed under the UK Home Office Licence 70/7185

Briefly, a population assay multigenerational experiment was used to cycle *P. berghei* malaria through mice and mosquitoes (Fig. 1; [12]). A statistical model was fitted to the observed data and experimental structure using a Bayesian posterior distribution in Stan [18]. The model predictions (posterior draws) for the parasite densities of respective life stages were then used to calculate the efficacy of ATV.

For each treatment regime (ATV-32% and control), mouse-to-mouse transmission operated as described previously [12, 13], the experiment is graphically demonstrated in Fig. 1 and described in the figure’s legend. All care and handling of animals strictly followed the Guidelines for Animal Care and Use prepared by Imperial College London, and was performed under the UK Home Office Licences 70/7185 and 70/8788.

**Fig. 2:**
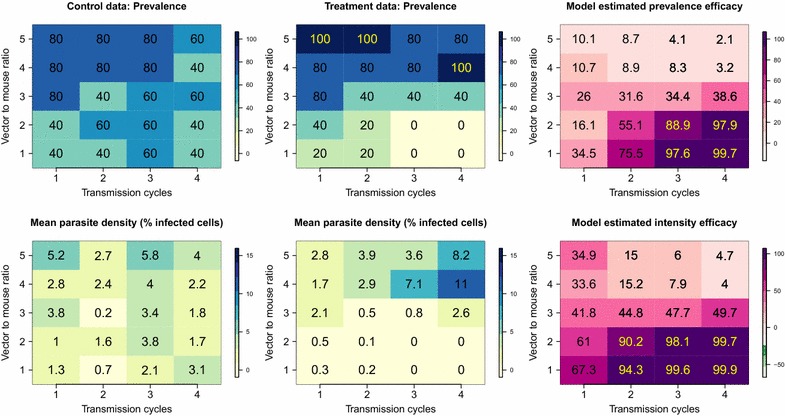
Summary of the observed experimental data and efficacy estimates against prevalence and parasite intensity. Prevalence (the percentage of infected individuals) and percentage parasite densities (the mean percentage of infected erythrocytes out of 1200 cells per host) in control (column 1) and treatment (column 2) mice for transmission cycles 1–4 (*x-axis*) and for biting rates 1–5 (1–5 mosquito bites per mouse,* y-axis*). Corresponding  % prevalence efficacy (*top right*) and % intensity efficacy (*bottom right*) estimated for each biting rate and transmission cycle using the probabilistic Bayesian model

Initial parasite density was measured by counting the number of infected red blood cells in the mice (*N* infected erythrocytes out of a total subsample of 1200 cells). Mosquitoes were dissected to assess the number of oocysts in the mosquito population. The sporozoite measurement was additionally binned into specified ranges (scores of 0–4 representing 0, 1–10, 11–100, 101–1000, 1000 + sporozoites, respectively). The structure of the data (from [12] and listed in Additional file 2) resulted in four complete scenarios whereby malaria was transmitted mouse-to-mouse via mosquitoes (Fig. 2).

### Statistical methods

In this experiment, it was not possible to measure the number of sporozoites reaching salivary glands from each oocyst or the number of blood-stage infections in mice resulting from injected sporozoites. These relationships are uncertain and are incorporated as nuisance parameters in the model (Additional file 1). Each of these stages is modelled sequentially, similarly to a hidden Markov model. The number of parasites in each stage is represented by a zero-inflated negative binomial distribution (NBD) to account for both infected and uninfected individuals. A bi-modal structure is fitted using a zero-inflation parameter, *π*, which determines the proportion of mice or mosquitoes that are uninfected and therefore cannot transmit. The shape of the relationships between different parasite life stages is unknown. This is accounted for by including a random effects component that allows the mean number of parasites in each group to vary according to the observed data. A comprehensive description of the model is provided in Additional file 1.

### Model fitting

All parameters were fitted jointly using a Bayesian posterior distribution in RStan (version 2.11, [18]). A non-centred parameterization method was employed [19, 20]. The model parameter fitting used a Hamiltonian Monte Carlo method [18], burn-in was 500 and the subsequent 500 samples from each chain (n = 4) were used for posterior predictive checks. The model R code is provided in Additional file 2), with the accompanying data (Additional file 2). The parameter estimates are supplied in Additional file 3.

### Model output

Two measures of efficacy are presented: (1) the TBA, the percentage difference in the proportion of infected hosts between the control (*t* = 0) and treatment (*t* = 1) arms of the experiment; and, (2) the TRA, the percentage difference in parasite density between the control and treatment arms of the experiment. Efficacy estimates (denoted *TBA*_*i,m*_ for prevalence, *TRA*_*i,m*_ for intensity), were generated for each biting rate (*m*) and transmission cycle (*i*) from the simulated posterior predictive model outputs (*n* iterations = 2000) using the equations: $$\begin{aligned} TBA_{i,m} = \frac{{P_{0,i,m} - P_{1,i,m} }}{{P_{0,i,m} }} \times 100 \hfill \\ TRA_{i,m} = \frac{{I_{0,i,m} - I_{1,i,m} }}{{I_{0,i,m} }} \times 100 \hfill \\ \end{aligned}$$.

The 95% credible intervals were calculated directly from the posterior predictive outputs (Table 1).

**Table 1:** Efficacy estimates

Group			Arithmetic mean (±1.96 × SE)		Median	
			TBA	TRA	TBA	TRA
Overall	N bites (m)	N cycles (*i*)	28.42 (28.0–28.9)	22.63 (21.7–23.6)	29.1	25.5
Per bite	1		28.42 (28.0–28.9)	22.63 (21.7–23.6)	29.1	25.5
	2		70.77 (69.8–71.7)	91.18 (90.7–91.6)	75.0	93.9
	3		58.60 (57.5–59.7)	88.58 (88.1–89.1)	62.5	91.2
	4		28.37 (27.2–29.5)	34.53 (32.9–36.1)	30.8	40.9
	5		5.87 (5.0–6.8)	−4.72 (−6.5 to −3.0)	6.3	−4.0
Per cycle		1	4.80 (3.9–5.7)	−8.78 (−10.6 to −7.0)	6.3	−12.2
		2	21.15 (20.3–22.0)	44.88 (43.8–45.9)	22.7	49.4
		3	30.20 (29.4–31.1)	28.63 (27.1–30.1)	31.3	35.0
		4	32.60 (31.7–33.5)	13.51 (11.7–15.3)	33.3	16.6
Per bite and cycle	1	1	28.42 (27.4–29.5)	4.16 (2.2–6.1)	28.6	1.3
	1	2	34.5 (32.0–37.0)	67.30 (65.7–68.9)	50.0	78.0
	1	3	75.53 (73.7–77.4)	94.33 (93.7–95.0)	100	100
	1	4	97.60 (97.0–98.2)	99.56 (99.3–99.8)	100	100
	2	1	99.70 (99.5–99.9)	99.9 (99.8–100)	100	100
	2	2	16.14 (13.4–18.9)	60.97 (59.3–62.6)	33.3	71.8
	2	3	55.14 (52.6–57.7)	90.18 (89.3–91.0)	66.7	96.9
	2	4	88.9 (87.6–90.2)	98.07 (97.7–98.5)	100	100
	3	1	97.93 (97.2–98.6)	99.74 (99.6–99.8)	100	100
	3	2	25.95 (23.8–28.1)	41.85 (39.8–43.9)	33.3	53.1
	3	3	31.60 (29.2–34.0)	44.80 (42.5–47.1)	33.3	57.5
	3	4	34.4 (31.7–37.2)	47.7 (45.3–50.2)	50.0	61.6
	4	1	38.60 (35.7–41.5)	49.7 (57.0–52.4)	50.0	66.3
	4	2	10.65 (8.9–12.4)	33.6 (31.6–35.6)	20.0	44.0
	4	3	8.88 (7.0–10.8)	15.18 (12.8–17.5)	0.0	22.1
	4	4	8.29 (6.2–10.4)	7.87 (5.3–10.4)	0.0	14.2
	5	1	3.23 (0.6–9.5)	4.00 (1.2–6.8)	0.0	9.5
	5	2	10.15 (8.6–77.4)	34.9 (33.0–36.9)	20.0	44.1
	5	3	8.65 (6.7–10.6)	14.98 (12.5–17.4)	0.0	21.8
	5	4	4.12 (1.59–6.7)	5.99 (3.3–8.7)	0.0	11.2

The mean and standard errors (±1.96 × SE) and median for prevalence, TBA and intensity efficacy, TRA estimates for biting rates 1–5 (the number of mosquito bites per mouse), transmission cycles 1–4, calculated from the posterior distributions of the probabilistic Bayesian model

*TBA* transmission-blocking activity, *TRA* transmission-reducing activity

To justify model assumptions, it was important to investigate the difference between the overall distribution of sporozoite scores in control and treatment groups, parasitaemia (%) and gametocytaemia (%). Data exploration was conducted in R, version 3.2.2 [21].

## Results

### Evaluating efficacy

With a full probabilistic model of the mouse-to-mouse measurements there is potential to define many possible measures of efficacy depending on exactly what elements of the model impact future interventions. Here two are presented: TBA and TRA (Table 1).

### The prevalence efficacy TBA

**Fig. 3:**
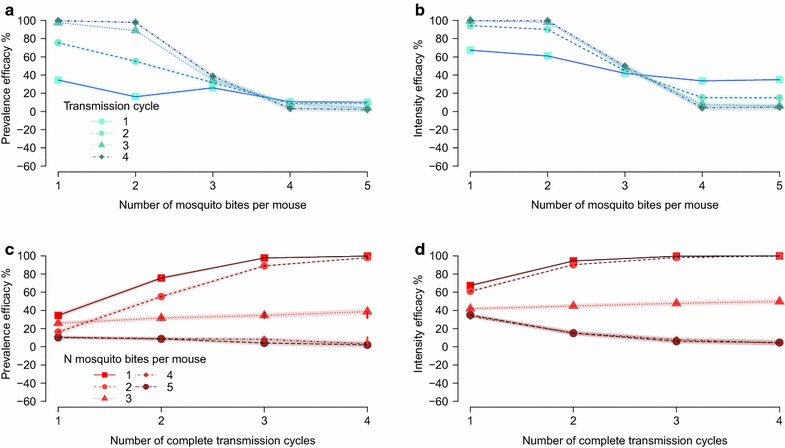
Impact of atovaquone 32% (ATV-32%) across transmission cycles and mosquito biting rates. The transmission-blocking (prevalence) (**a** and **c**) and transmission-reducing (intensity) (**b** and **d**) efficacy (%) and critical binomial 95% credible intervals (*shaded regions around lines*) generated from the model simulated treatment and control posterior distributions of the parasite density per individual for each biting rate (the number of mosquitoes that fed on each mouse, *row 1*) or transmission cycle (*row 2*)

The overall TBA of ATV-32% between the two arms of the experiment was 28.42% (28.0–28.9%: ±1.96 × standard error). There was a small increase in the impact against prevalence across transmission cycles (Table 1; Fig. 3) indicating multiple cycles were required to fully benefit from the ATV treatment.

The impact of ATV was greatest at low mosquito biting rates (where each mouse received a single mosquito bite, the TBA was 70.8% (69.8–71.7%); for two mosquito bites per mouse: 58.6% (57.5–59.7%); and, for three bites per mouse: 28.4% (27.2–29.5%)) whilst the impact was much reduced at higher mosquito biting rates [for four bites: 5.9% (5.0–6.8%); for five bites: 4.8% (3.9–5.7%)].

Per bite TBAs were consistent after the second completed transmission cycle (Fig. 3) suggesting that three cycles would be sufficient to capture the impact of ATV at a concentration expected to reduce transmission by 32% from vertebrate-to-vector [12].

### The intensity efficacy TRA

The overall TRA was 22.63% (21.7–23.6%). The biggest impact against intensity was achieved after the first transmission cycle (Table 1; Fig. 3).

Like parasite prevalence efficacies, the impact of ATV on parasite density was greatest at low mosquito biting rates; 91.2% (90.7–91.6%), 88.6% (88.1–89.1%) and 34.5% (32.9–36.1%) for one to three mosquito bites per mouse, respectively. There was no positive impact on parasite intensity at higher mosquito biting rates; −4.7% (−6.1–3.0%) and −8.8% (−10.6–7.0%) for four and five mosquito bites per mouse (Fig. 2).

**Fig. 4:**
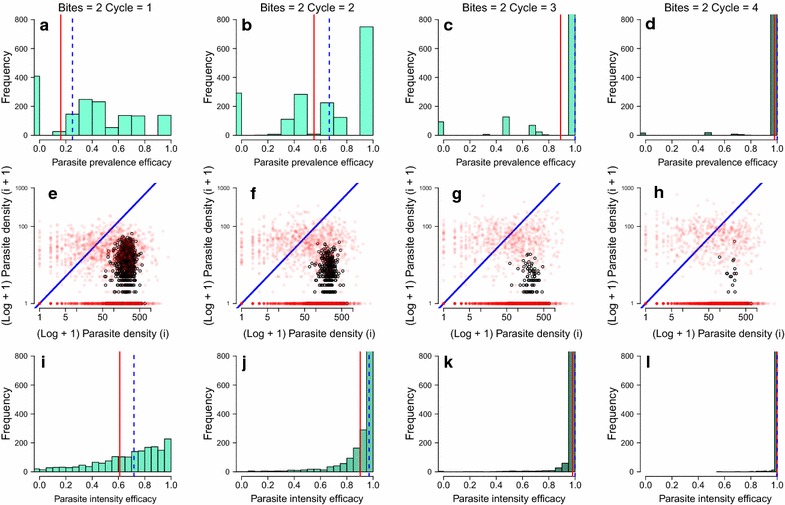
Comprehensive plots of the model output to determine efficacy. Each column presents successive transmission cycles 1, 2, 3 and 4 using the 2 mosquito bites per host group as an example. The generated posterior distributions from the model are the most probable values for parasite density for each of 2000 iterations. **a**–**d** Histograms of the estimated effect size for atovaquone 32% (ATV-32%) against prevalence (TBA) in the mice generated from the model posterior distributions. **e**–**h** The posterior predictive outputs for control (*red*) and treatment (*black*) data on the log scale + 1 for each transmission cycle (*i*). Where points fall below the blue line there has been a reduction in the parasitaemia from the initial (*i*) to the subsequent (*i* + *1*) cohort of hosts. The controls become both more numerous and dispersed in comparison to the treatment estimates as transmission progresses from transmission cycle 1–4; the drug has a positive impact both by reducing cases and by reducing the amount of infection through successive transmission cycles. **i**–**l** Histograms of the estimated effect size for ATV-32% against parasite density (TRA) in the mice generated from the model posterior distributions. For **a**–**d** and **i**–**l**, the *red solid vertical line* marks the mean estimate and the *dashed blue lines* indicate the median estimate

An illustration of the posterior outputs of the model is shown in Fig. 4.

### Parasite intensity, transmission and heavy infections

**Fig. 5:**
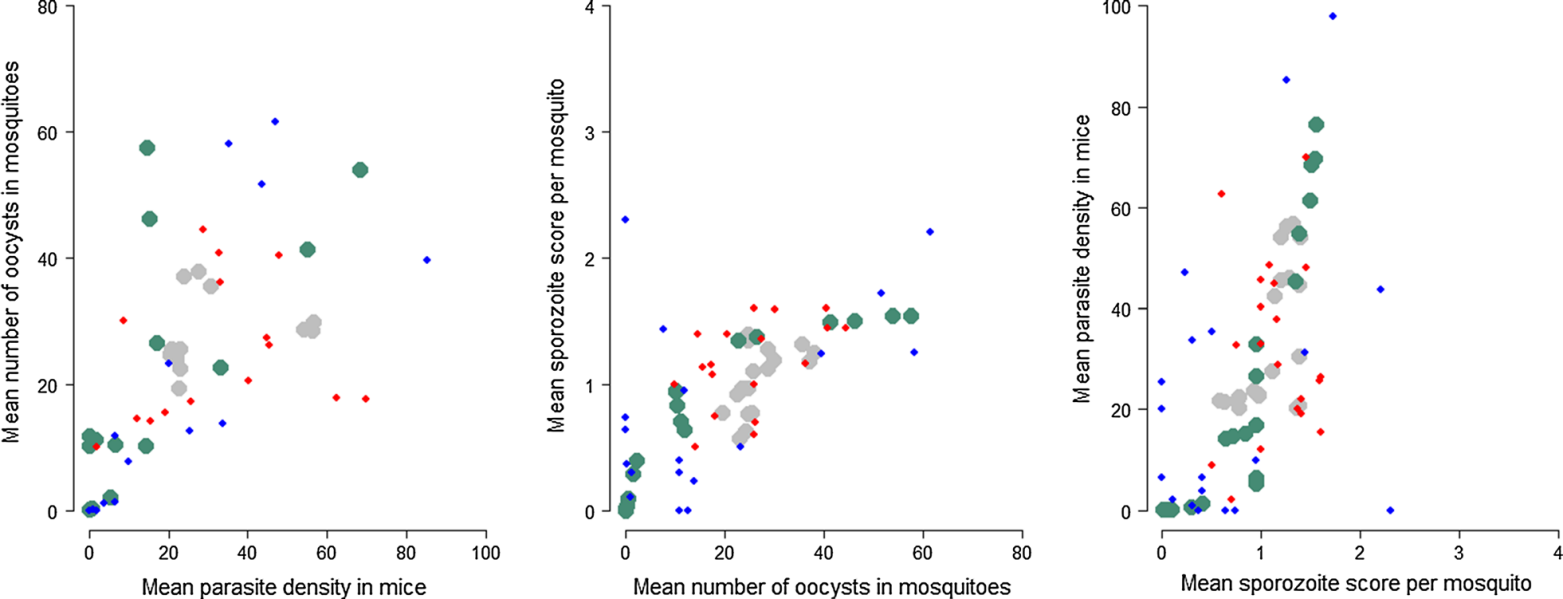
Relationships between malaria life stages. The mean estimates of the model (*grey*, control and *green*, treatment *dots*) and observed data (*red*, control and *blue*, treatment *dots*) describing relationships between successive life stages of malaria, demonstrating the broad agreement of data and model estimates

There is a non-linear relationship between each of the different parasite life stages in the model confirming that transmission between hosts and vectors (and vice versa) are saturating (negative) density-dependent processes (Fig. 5). Capturing these relationships enables more accurate estimation of efficacy by considering the natural population dynamics of infection that are also observed in the field for human infections [22].

Assessing efficacy estimates by transmission cycle and biting rate allows more detailed examination of the intervention’s impact. For example, in the treatment arm of the experiment, two of five mice in the transmission cycle 3 cohort, which received four mosquito bites each, had exceptionally high parasitaemia (≥12% of 1200 red blood cells were infected). Both mice had, by chance, been fed on by mosquitoes with very high post-feeding sporozoite counts (indicated by high mean sporozoite scores, >2). Once exposed, these mice can be fed upon by any of the 500 naïve mosquitoes in the next cohort. Consequently, higher than expected parasite densities are propagated throughout the system (Fig. 2).

Although the infectiousness of mice depends on the mature gametocytes present in the blood, rather than the asexual parasites (that are used in the model), there is less confidence in microscopic measurements of gametocyte counts. A linear relationship between mean gametocytaemia (the percentage of erythrocytes infected with sexual stage parasites) and mean parasitaemia (the percentage of erythrocytes infected with asexual stage parasites) was consistent with the data (*mean gametocytaemia* ~0.11 × *mean parasitaemia* + 0.03, *σ* = 0.12: F_40,38_ = 197.1, p < 0.001, _adj_R^2^ = 0.83), justifying the use of parasitaemia as a dependent variable in addition to infection status.

## Discussion

Accurate methods to assess potential TBIs are important to ensure that the best candidates go forward to expensive clinical trials. Here, TBI efficacy from mouse population assays is estimated by fitting a probabilistic Bayesian model to multiple parasitic life stages with a structure that reflects the experiment structure used to collect the data. Whilst it may be more computationally expensive to fit a multistage Bayesian model such as the one presented here, the method: (i) is able to identify chance events that can occur at any point in the experiment; (ii) is statistically robust to natural fluctuations in the starting parasite density, which impacts the capacity of the treatment to reduce secondary infections; and (iii) has the flexibility to capture the density dependent (or other) relationships between parasite life stages. The chain binomial model produced a relatively similar estimate for ATV-32% (20.4% reduction in secondary cases) when compared to the Bayesian approach (28.4% efficacy against prevalence).

Although the two metrics are not directly comparable, they provide complementary understanding of the intervention impact. Firstly, the chain binomial model presents an overall effect size that measures the reduction in secondary cases in hosts, the *R*_0_, that can be achieved by using vaccine. This can be used directly in mathematical models to understand the epidemiology of diseases. Conversely the Bayesian method directly calculates the reduction in the prevalence and density of infection between the different arms of the experiment. This generates more intuitive efficacy estimates which are more comparable to the results of clinical trials and are easier to understand by non-mathematical modellers. Secondly, the chain binomial model assumes that all infected mice and mosquitoes have the same infectivity irrespective of the density of infection. This can bias estimates depending on the quantity of parasite in the system. The new method allows the infectivity to vary according to the density of the parasite at the particular life-stages which provides additional information for the actions of an intervention. Finally, the chain binomial model generates a single effect size estimate whilst the Bayesian model enables intervention efficacy to be broken down by treatment round and biting rate. This finer scale of resolution allows a more detailed understanding of the dynamics of the disease, for example by showing that parasite density efficacy is higher in earlier transmission cycles compared to the efficacy against parasite prevalence. This finer resolution also enables chance fluctuations in parasite intensity to be identified, which could bias overall estimates in populations with small sample sizes and generate misleading results. One of the advantages of the new method is that it makes no assumptions about the likelihood of infection of multiply bitten hosts unlike the chain binomial model which assumes that mice are more likely to have detectable parasites the more infected bites they receive (following a binomial distribution). This makes results from the low biting groups relatively uncertain (due to stochastic fluctuations) so these groups should be interpreted in the context of other results.

Measuring impact at different mosquito biting rates and transmission cycles shows that ATV works first to reduce parasite density and later to reduce secondary infections. Single mouse-to-mouse transmission cycle population experiments have been performed previously [13]. Although this would generate accurate predictions of efficacy for highly effective interventions (which eliminate all onward transmission), using a single cycle of mouse-to-mouse transmission could potentially underestimate the impact of partially effective TBIs. The model also indicates that, for the intervention investigated here, efficacy estimates remain relatively stable after three cycles of transmission. This may allow the length of the experiment to be shortened, lowering costs and reducing the number of mice required.

The mouse population assay outlined in [12] is a standardized experiment and will be used to address future hypotheses including proof of concept questions such as the impact of combining interventions (for example a transmission-blocking vaccines with pre-erythrocytic vaccines) or changing levels of vaccine coverage. These types of experiments allow the consequences of different epidemiological characteristics or control interventions to be triaged in a controlled environment that would be infeasible to do in a human system or would require multiple, very expensive randomised control trials.

Given the relatively sparse data for each trial this analysis can be sensitive to systematic variation caused by fluctuations in the amount of malaria per individual in any given transmission cycle. The hierarchical structure used, however, does prevent a fluctuation in any one distribution (caused by unusually heavily infected individuals) from strongly biasing the inferences of other life-stages. Moreover, the weakly-informative priors used in the analysis limit the potential for overfitting and the predictive checks showed no indication of such pathologies. The analysis could be improved by incorporating greater biological realism into the model by increasing the number of intermediate life-stages (for example including a gametocyte life-stage or better capturing host immune responses). However, the benefit of this enhanced realism in the model depends on the applicability of the rodent system to human malaria. There is much debate about the capacity for mouse models to inform on aspects of human malaria [23–25] so extrapolating to *P. falciparum* must be done with caution.

## Conclusions

Here, a standardised statistical methodology is outlined which uses the Hamiltonian Monte Carlo method (RStan, version 2.11 [18]) to fit to population assay experimental data. To the author’s knowledge, this is the first time these methods have been used to examine parasitological data and their flexibility and precision makes them ideally suited for this purpose. In many parasitic infections the population dynamics of transmission and host morbidity and mortality depends on the number of parasites in a host and/or vector and not their presence/absence [26]. The distribution of parasites between hosts is typically highly aggregated [16, 27, 28]. This is particularly the case for many human parasitic infections, which are the target of elimination programmes [29, 30, 31]. As the parasite becomes increasingly rare, mean-based estimates of parasite intensity become increasingly uncertain. The range of statistical distributions available in Stan enables a more precise population estimate of parasite intensity. Many of the processes governing parasite transmission are density-dependent [27, 32]. Using a probabilistic approach enables these non-linear processes to be captured at the individual level whilst considering parasite distributions without the need for overly simplifying the experimental structure in the statistical model or running computationally expensive individual-based transmission models [33].

## Additional files

**Additional file 1.** A comprehensive statistical description of the probabilistic Bayesian model.

**Additional file 2.** A collection of files containing the original data in list format (Additional file 2.1), a ‘how to’ R script (Additional file 2.2) to apply the Bayesian model to DFA multi-generational data in RStan (Additional file 2.1 can be used as an example). The probabilistic Bayesian model code (Additional file 2.3) and the data (Additional file 2.1) used in Blagborough et al. [12] are supplied together with functions (Additional file 2.4) to help assess the model output (Additional file 2.5).

**Additional file 3.** The parameter estimates and the alternative efficacy measure using infection probabilities. Table 2.1 The table of parameter estimates and estimated mean values for the posterior predictive data in the model. Table 2.2 The ‘infection reduction’ efficacy $E^{R}_{i,m}$ is calculated as the difference between the summed mean *π*_*V*_ and mean *π*_*P*_ for controls compared to treatment arms for each transmission cycle *i* and biting rate *m.* The efficacy impact that can be attributed to *π*_*V*_ is shown.

## Abbreviations

TBI: transmission-blocking interventions
TBV: transmission-blocking vaccine
SMFA: standard membrane feeding assay
DMFA: direct membrane feeding assay
DFA: direct feeding assay
ATV: atovaquone
TBA: transmission-blocking activity
TRA: transmission-reducing activity
NBD: negative binomial distribution

## References

[1] WHO. World malaria report 2015. Geneva: World Health Organization, 2015. p. 243.

[2] Stone WJR, Eldering M, van Gemert G-J, Lanke KHW, Grignard L, van de Vegte-Bolmer MG (2013). The relevance and applicability of oocyst prevalence as a read-out for mosquito feeding assays. Sci Rep.

[3] Delves MJ, Ramakrishnan C, Blagborough AM, Leroy D, Wells TNC, Sinden RE (2012). A high-throughput assay for the identification of malarial transmission-blocking drugs and vaccines. Int J Parasitol.

[4] Sinden RE, Blagborough AM, Churcher T, Ramakrishnan C, Biswas S, Delves MJ (2012). The design and interpretation of laboratory assays measuring mosquito transmission of plasmodium. Trends Parasitol.

[5] Bousema T, Churcher TS, Morlais I, Dinglasan RR (2013). Can field-based mosquito feeding assays be used for evaluating transmission-blocking interventions?. Trends Parasitol.

[6] Stone WJR, Bousema T, Vaughan A (2015). The standard membrane feeding assay: advances using bioluminescence. Malaria vaccines: methods and protocols.

[7] Matsuoka H, Kobayashi J, Barker GC, Miura K, Chinzei Y, Miyajima S (1996). Induction of anti-malarial transmission blocking immunity with a recombinant ookinete surface antigen of *Plasmodium berghei* produced in silkworm larvae using the baculovirus expression vector system. Vaccine.

[8] Blagborough AM, Delves MJ, Ramakrishnan C, Lal K, Butcher G, Sinden RE, Menard R (2013). Assessing transmission-blocking in *Plasmodium* spp. Malaria methods and protocols. Methods in molecular biology.

[9] Churcher TS, Blagborough AM, Delves M, Ramakrishnan C, Kapulu MC, Williams AR (2012). Measuring the blockade of malaria transmission—an analysis of the standard membrane feeding assay. Int J Parasitol.

[10] Bolker BM, Brooks ME, Clark CJ, Geange SW, Poulsen JR, Stevens MH (2008). Generalized linear mixed models: a practical guide for ecology and evolution. Trends Ecol Evol.

[11] RTSS Clinical Trials Partnership (2015). Efficacy and safety of RTS, S/AS01 malaria vaccine with or without a booster dose in infants and children in Africa: final results of a phase 3, individually randomised, controlled trial. Lancet.

[12] Blagborough AM, Churcher TS, Upton LM, Ghani AC, Gething PW, Sinden RE (2013). Transmission-blocking interventions eliminate malaria from laboratory populations. Nat Commun.

[13] Upton LM, Brock PM, Churcher TS, Ghani AC, Gething PW, Delves MJ (2015). Lead clinical and preclinical antimalarial drugs can significantly reduce sporozoite transmission to vertebrate populations. Antimicrob Agents Chemother.

[14] Greenwood M (1931). On the statistical {measure} of infectiousness. J Hyg (Lond).

[15] Becker N (1981). A general chain binomial model for infectious diseases. Biometrics.

[16] Sinden RE, Dawes EJ, Alavi Y, Waldock J, Finney O, Mendoza J (2007). Progression of *Plasmodium berghei* through *Anopheles stephensi* is density-dependent. PLoS Pathog.

[17] Pichon G, Awono-Ambene HP, Robert V (2000). High heterogeneity in the number of *Plasmodium falciparum* gametocytes in the bloodmeal of mosquitoes fed on the same host. Parasitology.

[18] Stan Development Team. Stan modeling language users guide and reference manual. 2016.

[19] Papaspiliopoulos O, Roberts GO, Sköld M (2007). A general framework for the parametrization of hierarchical models. Stat Sci.

[20] Betancourt M, Girolami M, Upadhyay SK, Singh U, Dey DK, Logananthan A (2015). Hamiltonian Monte Carlo for hierarchical models. Current trends in Bayesian methodology with applications.

[21] R Core Team. R: A language and environment for statistical computing. Vienna: R Foundation for Statistical Computing; 2014. http://www.r-project.org/.

[22] Slater HC, Ross A, Ouédraogo AL, White LJ, Nguon C, Walker PGT (2015). Assessing the impact of next-generation rapid diagnostic tests on *Plasmodium falciparum* malaria elimination strategies. Nature.

[23] Wykes MN, Good MF (2009). What have we learnt from mouse models for the study of malaria?. Eur J Immunol.

[24] Fidock DA, Rosenthal PJ, Croft SL, Brun R, Nwaka S (2004). Antimalarial drug discovery: efficacy models for compound screening. Nat Rev Drug Discov..

[25] Langhorne J, Buffet P, Galinski M, Good M, Harty J, Leroy D (2011). The relevance of non-human primate and rodent malaria models for humans. Malar J.

[26] Anderson RA, May RM (1991). Infectious diseases in humans: dynamics and control.

[27] Shaw DJ, Dobson AP (1995). Patterns of macroparasite abundance and aggregation in wildlife populations: a quantitative review. Parasitology.

[28] Woolhouse ME, Dye C, Etard JF, Smith T, Charlwood JD, Garnett GP (1997). Heterogeneities in the transmission of infectious agents: implications for the design of control programs. Proc Natl Acad Sci USA.

[29] Galvani AP, May RM (2005). Epidemiology: dimensions of superspreading. Nature.

[30] Linehan M, Hanson C, Weaver A, Baker M, Kabore A, Zoerhoff KL (2011). Integrated implementation of programs targeting neglected tropical diseases through preventive chemotherapy: proving the feasibility at national scale. Am J Trop Med Hyg.

[31] Anderson RM, Truscott JE, Pullan RL, Brooker SJ, Hollingsworth TD (2013). How effective is school-based deworming for the community-wide control of soil-transmitted helminths?. PLoS Negl Trop Dis.

[32] Shaw DJ, Grenfell BT, Dobson AP (1998). Patterns of macroparasite aggregation in wildlife host populations. Parasitology.

[33] Churcher TS, Filipe JAN, Basáñez MG (2006). Density dependence and the control of helminth parasites. J Anim Ecol.

