# Supplementary material for: A novel model fitted to multiple life stages of malaria for assessing efficacy of transmission-blocking interventions
Source: Malar J. 2017 Apr 4;16:137. doi: 10.1186/s12936-017-1782-3 (PMC5379616; doi:10.1186/s12936-017-1782-3)
Supplement: Supplementary file 1 — Additional file 1. A comprehensive statistical description of the probabilistic Bayesian model. [file 12936_2017_1782_MOESM1_ESM.pdf]

A novel model fitted to multiple life stages of malaria for assessing efficacy of transmission-blocking interventions

Ellie Sherrard-Smith[a*], Thomas S. Churcher[a], Leanna M. Upton[b], Katarzyna A. Sala[b], Sara E. Zakutansky[b], Hannah C. Slater[a], Andrew M. Blagborough[b], Michael Betancourt[c]

Supplementary Information 1: Description of the model

The data used in this analysis is from a direct feeding assay (DFA) described in Fig. 1 and [1]. A statistical model was fitted to the observed data and experimental structure using a Bayesian posterior distribution in Stan [2]. The model predictions (posterior draws) for the parasite densities of respective life stages were then used to calculate the efficacy of ATV.

For each treatment regime (ATV-32% and control), mouse-to-mouse transmission operated as described previously [1, 3], the experiment is graphically demonstrated in Fig. 1 and described in the figure legend. All care and handling of animals strictly followed the Guidelines for Animal Care and Use prepared by Imperial College London, and was performed under the UK Home Office Licences 70/7185 and 70/8788.

Initial parasite density was measured by counting the number of infected red blood cells in the mice (*N* infected erythrocytes out of a total subsample of 1200 cells). Mosquitoes were dissected to assess the number of oocysts in the mosquito population. The sporozoite measurement was additionally binned into specified ranges (scores of 0-4 representing 0, 1-10, 11-100, 101-1000, 1000+ sporozoites,

respectively). The structure of the data (from [1] and listed in S2.1) resulted in 4 complete scenarios whereby malaria was transmitted mouse-to-mouse via mosquitoes.

Statistical methods

The data consist of measurements of the life stages in mosquito midguts, salivary glands and mice. In this experiment, it was not possible to measure the number of sporozoites reaching the salivary glands from each oocyst or the number of blood-stage infections in mice resulting from injected sporozoites. These relationships are uncertain and are incorporated as nuisance parameters in the model. Each of these stages are modelled sequentially, similarly to a hidden Markov model. The number of parasites in each stage is represented by a zero-inflated

negative binomial distribution to account for both infected and uninfected individuals. A bi-modal structure is fitted using a zero-inflation parameter, π. This parameter determines the proportion of mice or mosquitoes that are uninfected and therefore cannot transmit. The shape of the relationships between different parasite life stages are unknown. This is accounted for by including a random effects component that allows the mean number of parasites in each group to vary according to the observed data.

Seeding mouse population

Let each treatment arm (t = 0 for controls, 1 for ATV treatment) inform the mean μ and dispersion φ parameters for the distribution of parasite densities (N infected erythrocytes out of 1200 cells). At the start of the experiment (transmission cycle $i = 0$), μ and φ describe the parasite densities in mice N_0 that have been injected with high numbers of parasites. A negative binomial distribution is fitted to the initial mice

population for each treatment arm and these are convoluted into a global distribution as there is no biological reason for treatment arms to have different numbers of injected parasites at this stage, therefore,

$$N_0 \sim NB(\mu, \varphi).$$

Throughout, the negative binomial is parameterised by a mean parameter and a parameter that controls for overdispersion of the variance relative to the square of the mean (described as neg_binomial_2 in the RStan manual, [2]). The model parameters are fit on the log scale, the data are kept as linear counts. After the initial mice stage, the transmission is looped through the respective mosquito to mouse cycles for as many cycles *i* as there are data, for each control or treatment arm. At each subsequent life stage a new estimate of μ and φ describe the parasite density.

Oocyst counts in mosquitoes

In the experiment, the mosquito population becomes infected by feeding on all 5 mice simultaneously; any mosquito could feed on any mouse. Therefore, there is no effect of biting rate for the cohort of mosquitoes that all feed on all mice. Following the experimental design (Fig. 1), the variation that is observed between biting rates (m = 1 to 5 mosquito bites per mouse) is naturally introduced when a sub-sample of a pre-determined number of mosquitoes are randomly selected to feed on each mouse (m = 1 to 5 mosquito bites per mouse) at the second transmission cycle (i = 1), and onward. (This is reflected in the Bayesian model below by having random effects for each biting rate inform the distribution of parasites in mice for the sporozoite to mouse transmission step.) Let the number of oocysts in each mosquito with infection (O') be described by a negative binomial distribution,

$$O' \sim NB(o_{imt}, \tau_{imt})$$

Where o_{imt} and τ_{imt} are the mean and dispersion parameters defining the population of oocysts in mosquitoes for each transmission cycle *i,* biting rate *m* and treatment *t*. The o_{imt} and τ_{imt} parameters for each transmission cycle are estimated using the parasite density in the previous life stage of the transmission cycle, *i* - 1:

$$\log(o_{imt}) = A_t \log(\mu_{(i-1)mt}) + R_t \log(\varphi_{(i-1)mt}) + (\alpha_{im0} - J_t)$$

$$\log(\tau_{imt}) = B_t \log(\mu_{(i-1)mt}) + T_t \log(\varphi_{(i-1)mt}) + \beta_t$$

Here, A_t, B_t, R_t and T_t are used to adjust the mean numbers of parasites in the mice to the mean number of oocysts in the mosquito (an average for all transmission cycles and biting rates), taking into account the mean and dispersion parameter seen in the mouse population (both of which contribute to the distribution of parasites in the next generation). Separate values of A_t, B_t, R_t and T_t are estimated for the control and interventions group and the difference in parameters indicates the average (per transition) impact of the TBI. The random effects component α_{im0} accounts for the non-linearity in the transition between life stages and any difference in efficacy between biting groups and across transmission cycles (α_{im0} is normally distributed and centred around zero for the control group). The random effect β_t is assumed to have a weakly-informative normally distributed prior.

Parameter J is incorporated to help the fitting process and takes a different value for each intervention tested. Here it is set at the transmission reduction efficacy of ATV as measured in a standard membrane feeding assay (where $(1 - J)_t$ equals 0 in the control arm, *t* = 0, and 0.68 in the intervention arm, *t* = 1).

The probability of onward infection in mosquitoes depends on whether the observed mouse was infected or not. For the first transmission cycle, where the parasite moves from the injected mice to mosquitoes, all mice are assumed to be infected. For subsequent transmissions, the zero-inflation parameter (either mice π_P or mosquitoes π_V) ensures no onward transmission from uninfected individuals. As progressively fewer individuals have infection in the treatment arm, the probability of onward transmission decreases for each group (aggregated by transmission cycle *i*, biting rate *m* and treatment *t*). The π parameter is informed by γ_{imt}, the probability of not transmitting infection onward given infection:

$$\text{logit}(\gamma_{imt}) = C_t \log(\mu_{(i-1)mt}) + U_t \log(\varphi_{(i-1)mt}) + \varepsilon_{imt}$$

Where;

$$\pi_V = P(\text{not infected mosquito}|\text{not infected mouse})\pi_P + P(\text{not infected mosquito}|\text{infected mouse})(1 - \pi_P)$$

$$\pi_V = 1 * \pi_P + P(\text{not infected mosquito}|\text{infected mouse})(1 - \pi_P)$$

$$\pi_V = \pi_P + \gamma_{imt}(1 - \pi_P)$$

The prior estimates for the coefficients C_t and U_t and random effect ε_{imt} have weakly-informative normally distributed priors for each treatment arm *t*. The probability of mosquitoes having oocysts is sampled from a mixture of the uninfected and infected distributions. If the parasite count in the previous mouse is zero, then this probability is informed by π_V, otherwise by 1 – π_V.

$$P(O_{imt}|\pi_V, o_{imt}, \tau_{imt}) = \pi_V \delta + (1 - \pi_V) NB(O_{imt}|o_{imt}, \tau_{imt})$$

Where δ is logical and depends on whether the predicted parasite density in mice *N* is zero;

$$\delta = \begin{cases} 1, & if\ N = 0 \\ 0, & if\ N \neq 1 \end{cases}$$

(and see neg_binomial_2 in the RStan manual, [2]). Here, O_{imt} are the measured data on whether a mosquito has oocysts (1) or not (0) and o_{imt} and τ_{imt} are parameters of the latent distribution.

Sporozoite counts in mosquitoes

A sub-sample of the mosquito population is randomly selected to infect the next generation of mice. The number of sporozoites remaining in the salivary glands following blood-feeding (as measured by microscopy) is called residual-sporozoite score, S_{imt}. It is recorded in bins on the logarithmic scale (number of bins = 5; 1 represents mosquitoes with no sporozoites, 2 is for 1-10 sporozoites, 3 for 11-100 sporozoites, 4 for 101-1000 sporozoites and 5 for more than 1000 sporozoites) resulting in a multinomial distribution. The mosquitoes dissected to determine residual-sporozoite score are sampled from the same cohort of mosquitoes that are sampled to determine the number of oocysts. Therefore, there is no need to adjust for TBI treatment as this is already incorporated during the parasite to oocysts transition. The mean s_{imt} and dispersion σ_{imt} parameters describe the sporozoite distributions in mosquitoes and are estimated using the o_{imt} and τ_{imt} parameters describing the oocysts in the previous stage,

$$\log(s_{imt}) = D\log(o_{(i-1)mt}) + V\log(\tau_{(i-1)mt}) + \omega_{im} + A$$

$$\log(\sigma_{imt}) = E\log(o_{(i-1)mt}) + W\log(\tau_{(i-1)mt}) + \epsilon_{im}$$

Here, a constant offset A is used to adjust for the increase in parasite life stages between the oocyst counts and the salivary gland sporozoites (on average, 1,250

(interquartile range 313 – 2,400) salivary gland sporozoites are released from a single *Plasmodium falciparum* oocyst [4]). The effect of TBI treatment is already present in the mosquito population so the coefficients *D,* E, *V* and *W* are the same for all intervention groups. These parameters and the random effects ω_{im} and ϵ_{im} are assumed to have weakly-informative normally distributed priors.

The probability of sporozoites in a given bin, *b*, will be equal to the cumulative distribution frequency (cdf) up to that bin (*b*) minus the cdf up to the preceding bin (*b* - 1), for example:

$$cdf(S_{imt_b}) \sim NB\left(S_{imt_b} | s_{imt}, \sigma_{imt}\right)$$

$$P(b_1) = \pi_V + (1 - \pi_V) NB_{cdf}(b_1 | s_{imt}, \sigma_{imt})$$

$$P(b_3) = (1 - \pi_V)\left[NB_{cdf}(b_3 | s_{imt}, \sigma_{imt}) - NB_{cdf}(b_2 | s_{imt}, \sigma_{imt})\right]$$

The sporozoite count in mosquitoes is then estimated as:

$$S_{imt} \sim Multinomial(b, S_{imt})$$

1.1.1 Parasite density in mice

Like the transmission of infection from mice to mosquitoes, the probability of infections in mice depends on whether the observed mosquito (dissected to determine residual-sporozoite score) was infected. This time, π_P parameter is informed by ζ_{imt}, the probability of not transmitting infection onward to mice given infection in the mosquito:

$$\text{logit}(\zeta_{imt}) = F_{mt} \log(o_{(i-1)mt}) + X_{mt} \log(\tau_{(i-1)mt}) + \kappa_{imt}$$

Where (derived as for π_V);

$$\pi_P = \pi_V + \zeta(1 - \pi_V)$$

The probability that a mouse will become infected depends on the number of mosquito bites it receives (m = 1 to 5 mosquito bites per mouse). Pre-erythrocytic vaccines upregulate antibodies that can inhibit sporozoites and therefore reduce the probability that an infected mosquito bite will result in infection. Though no PEV is used in this experiment, the possible impact is added to the model structure allowing analysis of these types of data in the future. Therefore, the coefficients F_{mt} and X_{mt} take different values for each biting rate m and treatment arm t. (Parameters A_t, B_t, R_t and T_t above do not vary between biting rate group to mimic the experimental set up).

Let the number of infected cells in the blood sample of 1200 erythrocytes (parasite density) in each mouse (N) be described by a negative binomial distribution,

$$N_{imt} \sim NB(\mu_{imt}, \varphi_{imt})$$

Where μ_{imt} and φ_{imt} are the mean and dispersion parameters defining parasite intensity in mice. After transmission cycle i = 0, the parameters are estimated using the sporozoite counts of malaria infection in the previous transmission cycle:

$$\log(\mu_{imt}) = G_{mt} \log(s_{(i-1)mt}) + Y_{mt} \log(\sigma_{(i-1)mt}) + \varsigma_{imt} + AP$$

$$\log(\varphi_{imt}) = H_{mt} \log(s_{(i-1)mt}) + Z_{mt} \log(\sigma_{(i-1)mt}) + \psi_{imt}$$

Again, the constant offset AP adjusts for a change in parasite life stages between the sporozoite counts and parasite densities measured in mice. Parameters G_{mt}, Y_{mt}, H_{mt} *and* Z_{mt}, have weakly-informative normally distributed priors for each biting rate m and treatment t. The random effect components ς_{imt} and ψ_{imt} are centred on zero and

allow the relationship between sporozoite score and asexual parasitemia to be determined by these data.

The probability of infections in mice is a mixture of the uninfected and infected distributions and informed by the incremental log probability. So that:

$$P(N_{imt}|\pi_P, \mu_{imt}, \varphi_{imt}) = \pi_P\delta + (1 - \pi_P)NB(N_{imt}|\mu_{imt}, \varphi_{imt})$$

Where,

$$\delta = \begin{Bmatrix} 1, & if\ N = 0 \\ 0, & if\ N \neq 1 \end{Bmatrix}$$

and N_{imt} indicates where mice do (1) or do not (0) have parasitic infection.

Model fitting

All parameters were fitted jointly using a Bayesian posterior distribution in RStan (version 2.13.1, [2]). To ensure robust fits, a non-centred parameterisation method was employed [5, 6]. The model parameter fitting was achieved using a Hamiltonian Monte Carlo method [2], burn-in was 500 and the subsequent 500 samples from each chain (n = 4) were used for the posterior predictive checks. The model code that can be used in R is provided in supporting information S2, with the accompanying data (S2.1). The parameter estimates are supplied in S3.

2.3 Model output

Here, 2 different measures of efficacy are presented (and see S3); 1) the transmission blocking efficacy TBE, the percentage difference in the proportion of infected hosts between the control ($t = 0$) and treatment ($t = 1$) arms of the experiment, and; 2) the transmission reduction efficacy TRE, the percentage

difference in parasite density between the control and treatment arms of the experiment. Efficacy estimates (denoted $TBE_{i,m}$ for prevalence, $TRE_{i,m}$ for intensity), were generated for each biting rate (*m*) and transmission cycle (*i*) from the simulated posterior predictive model outputs (*n* iterations = 2000) using the equations:

$$TBE_{i,m} = \frac{P_{0,i,m} - P_{1,i,m}}{P_{0,i,m}} \times 100 \qquad TRE_{i,m} = \frac{I_{0,i,m} - I_{1,i,m}}{I_{0,i,m}} \times 100$$

The 95% credible intervals were presented as 1.96 x the standard error for the efficacy estimates. These were calculated directly from the posterior predictive outputs (Table 1).

To justify model assumptions, it was important to investigate the difference between the overall distribution of sporozoite scores in control and treatment groups, parasitemia (%) and gametocytemia (%). Data exploration was conducted in R, version 3.2.2 [7].

References

1. Blagborough AM, Churcher TS, Upton LM, Ghani AC, Gething PW, Sinden RE. Transmission-blocking interventions eliminate malaria from laboratory populations. Nat Comms. 2013;4: 1812. doi: 10.1038/ncomms2840
2. Stan Development Team. Stan Modeling Language Users Guide and Reference Manual. Version 2.13.1 {http://c-stan.org/} 12th Dec 2016.
3. Upton LM, Brock PM, Churcher TS, Ghani A, Gething PW, Delves MJ, et al. Lead clinical and preclinical antimalarial drugs can significantly reduce

sporozoite transmission to vertebrate populations. Antimicrobial Agents and Chemotherapy 2015;59: 490-497. doi: 10.1128/AAC.03942-14

4. Stone WJ, Eldering M, van Genert GJ, Lanke KH, Grignard L, van de Vegte-Bolmer MG, et al. The relevance and applicability of oocyst prevalence as a read-out for mosquito feeding assays. Scientific Reports 2013;3: 3418. doi: 10.1038/srep03418
5. Papaspiliopoulos O, Roberts GO, Sköld M. A general framework for the parameterization of hierarchical models. Statistical Science 2007;22: 59-73.
6. Betancourt M, Girolami M. Hamiltonian Monte Carlo for Hierarchical Models. In: Upadhyay SK, Singh U, Dey DK, Logananthan A, editors. Current Trends in Bayesian Methodology with Applications. Chapman and Hall/CRC Press; 2015. pp. 680. ISBN 9761482235111
7. R Core Team. R: A language and environment for statistical computing. R Foundation for Statistical Computing, Vienna, Austria. URL {http://www.R-project.org/} 2014.
8. Blagborough AM, Delves MJ, Ramakrishnan C, Lal K, Butcher G, Sinden RE. In: Malaria Methods and Protocols. Chapter 7.6 (Ed. Menard R) Assessing transmission-blocking in Plasmodium spp. Humana, 2012. pp.577–600.
